# A novel Hsp70 inhibitor prevents cell intoxication with the actin ADP-ribosylating *Clostridium perfringens* iota toxin

**DOI:** 10.1038/srep20301

**Published:** 2016-02-03

**Authors:** Katharina Ernst, Markus Liebscher, Sebastian Mathea, Anton Granzhan, Johannes Schmid, Michel R. Popoff, Heiko Ihmels, Holger Barth, Cordelia Schiene-Fischer

**Affiliations:** 1 Institute of Pharmacology and Toxicology, University of Ulm Medical Center, Ulm, Germany; 2 Max Planck Research Unit for Enzymology of Protein Folding Halle, Halle (Saale), Germany; 3 Department of Chemistry and Biology, University of Siegen, Germany; 4 Department of Anaerobic Bacteria, Pasteur Institute, Paris, France

## Abstract

Hsp70 family proteins are folding helper proteins involved in a wide variety of cellular pathways. Members of this family interact with key factors in signal transduction, transcription, cell-cycle control, and stress response. Here, we developed the first Hsp70 low molecular weight inhibitor specifically targeting the peptide binding site of human Hsp70. After demonstrating that the inhibitor modulates the Hsp70 function in the cell, we used the inhibitor to show for the first time that the stress-inducible chaperone Hsp70 functions as molecular component for entry of a bacterial protein toxin into mammalian cells. Pharmacological inhibition of Hsp70 protected cells from intoxication with the binary actin ADP-ribosylating iota toxin from *Clostridium perfringens*, the prototype of a family of enterotoxins from pathogenic Clostridia and inhibited translocation of its enzyme component across cell membranes into the cytosol. This finding offers a starting point for novel therapeutic strategies against certain bacterial toxins.

Assistance of folding and restructuring of polypeptide chains is the main feature of folding helper proteins that also provide support for the maintenance of correctly folded client proteins in the cell. In some cases a network of folding helper proteins comprising peptidyl prolyl *cis/trans* isomerases (PPIases) and heat shock proteins (Hsps) assemble as multimeric complexes allowing for coordinated interactions with client proteins like steroid hormone receptors, the tumor suppressor p53, the RNA-induced silencing complexes that contain AGO proteins and single-stranded small RNAs or ion channels like the voltage-dependent delayed rectifier potassium channel (HERG) or the cystic fibrosis transmembrane conductance regulator (CFTR)^1^,^2^,^3^,^4^,^5^,^6^,^7^. For the major players of the PPIase families, the cyclophilin and FK506-binding proteins (FKBPs), cyclosporins and FK506 derivatives, respectively, serve as high affinity, low molecular mass inhibitors of the PPIases function of these enzymes^8^,^9^. Thus, they offer versatile tools to assess the physiological role of the protein folding network in living cells providing a clear indication for the chemical mode of action of these enzymes in the cell^4^,^10^,^11^. Due to the lack of specific small molecule inhibitors, the Hsp70 family of folding helpers, which is particularly known for stress protection, appears to be less amendable to a similar pharmacological approach.

Hsp70 proteins form a conserved family of molecular chaperones. They consist of an N-terminal nucleotide binding domain of approximately 44 kDa linked to an about 25 kDa C-terminal substrate binding domain and are unique because, as exemplified by the *E.coli* Hsp70 protein DnaK, two catalytic domains make up their functional features. The ATPase domain is controlled by ATP binding as nucleotide binding induces structural rearrangements in the ATPase as well as in the substrate binding domain which allow interdomain communication and promote high on-off rates for the substrate^12^,^13^. The substrate binding domain transiently interacts with exposed regions of a multitude of partially folded or unfolded substrate proteins to exert its chaperone function to promote and regulate protein folding. The bacterial Hsp70 protein DnaK was identified as a secondary amide peptide bond *cis/trans* isomerase (APIase), which selectively accelerates the *cis/trans* isomerization of non-proline peptide bonds^14^. This activity resides in the substrate binding domain and is considered to assist folding processes by increasing the peptide chain flexibility around the rigid secondary amide functionality. Like DnaK, its human orthologs Hsp70 and Hsc70 form key components in the folding and maintenance of functional proteins in the cell.

Human Hsp70s are regarded as a cellular protective system against cellular stress and thus are critical for cell survival. Also, Hsp70 proteins are involved in the control of the biological activity of a large number of client proteins like steroid hormone receptors, kinases and transcription factors. Although there is much to be discovered about the role of Hsp70s it is already known that they are involved in cell cycle regulation, signal transduction, and apoptosis^15^,^16^. Importantly, Hsp70 helps to mediate protein translocation across membranes. For example, the mitochondrial Hsp70 mediates the translocation of polypeptides into mitochondria by acceleration of unfolding and entropic pulling^17^.

Notably, studies on the uptake and intracellular membrane transport of various medically relevant bacterial ADP-ribosylating toxins using pharmacological inhibitors of PPIases and heat shock protein 90 (Hsp90) indicated that these host cell factors play an important role in the uptake of the enzymatically active subunits of these toxins into the host cell cytosol^18^,^19^,^20^,^21^,^22^,^23^,^24^,^25^,^26^,^27^,^28^. In fact, the targeted pharmacological inhibition of individual host cell chaperones/PPIases thus prevented the translocation of the toxins into the cytosol and protected cells from intoxication. The composition of other multimeric complexes of folding helper proteins is suggestive of an Hsp70 participation in the toxin transport pathway in our opinion. Unfortunately, the direct identification of this role of Hsp70 has been hampered by the lack of specific Hsp70 inhibitors.

Previously, proline-rich pyrrhocoricin-derived oligopeptides were shown to target the substrate binding domain of DnaK thereby interfering with its APIase activity^29^. Reduction of the molecular mass of this type of APIase inhibitor could be achieved by developing a series of fatty acylated (4-aminoalkylbenzoyl)-L-amino acids^30^. The detergent nature of the fatty acylated inhibitors has prevented the characterization of the weak inhibition of Hsp70 *in vitro*. Therefore, the development of a novel inhibitor was demanded to investigate the mode of action of Hsp70 in more detail.

Principally, the coupling between both domains of Hsp70s, the ATPase and the substrate binding domain, should provide a good opportunity to block Hsp70 functions by low molecular weight inhibitors directed against the ATPase active site. Thus, in search of inhibitors of Hsp70s a structure-based design study has identified submicromolar affinity binders of the ATP binding pocket such as 3′-sulfogalactolipids^31^,^32^. Additionally, screening identified inhibitors of the ATPase activity of DnaK that share a dihydropyrimidine core^33^. Another structure-based approach starting from adenosine identified the small molecule VER-155008 which binds to the ATP binding pocket of Hsp70 with a dissociation constant of 0.3 μM^34^. It also inhibits other members of the Hsp70 family as demonstrated for Hsc70 and the endoplasmic reticulum Hsp70 Grp78^35^.

However, Hsp70s are highly dynamic and conformational transitions connected with the substrate affinity also occur independent from the nucleotide status of the protein^13^. Additionally, Hsp70 lacking the ATPase domain is able to provide some level of protection of cells against thermal stress and was shown to reduce heat-induced nuclear protein aggregation, although the ATPase-deleted protein is less effective as the wild-type Hsp70^36^,^37^. Therefore, targeting the Hsp70 substrate binding domain by small molecules forms an attractive alternative to completely block its chaperoning activity. For human Hsp70, 2-phenylethynesulfonamide (PES) was found to represent an inhibitor of its chaperone function^38^. It inhibits the Hsp70-assisted refolding of firefly luciferase^39^. Initially it was shown that Hsc70 or Grp78 are not able to interact with PES^38^. Later on it was found that PES is not specific for a Hsp70 isoform and it appears to exhibit no affinity to the peptide binding site of Hsp70^39^,^40^. Taken together, up to now there is no specific inhibitor available that directly targets the substrate binding domain of human Hsp70.

In conclusion, targeting Hsp70 family proteins by small molecules is of great interest, because members of this family are involved in a wide variety of cellular pathways and interact with a large number of different proteins, including key factors in signal transduction, transcription, cell-cycle control, stress response and membrane transport. In this study we have identified the stress-inducible chaperone Hsp70 to function as molecular component of the entry route of the binary actin ADP-ribosylating iota toxin from *Clostridium (C.) perfringens*. Pharmacological inhibition of Hsp70 prevented the pH-driven translocation of the enzyme component of this toxin across cell membranes into the cytosol thereby protecting cells from intoxication. This was achieved by the development of a new Hsp70 low molecular weight inhibitor specifically targeted to the peptide binding site of Hsp70. Evaluation of the properties of the small molecule inhibitor indicated inhibition of *in vitro* Hsp70-mediated folding assistance as well as *in vivo* increasing the sensitivity of cells to apoptosis.

## Results

### Characterization of the Hsp70 inhibition by compound 1

To identify a selective small-molecule inhibitor of the APIase site of Hsp70, a compound library was screened for inhibition of the Hsp-assisted refolding of denatured firefly luciferase *in vitro.* This effort has led to the identification of the 9-aminoacridizinium derivative **1** as an inhibitor of the Hsp70 foldase function.

**Figure 1:**
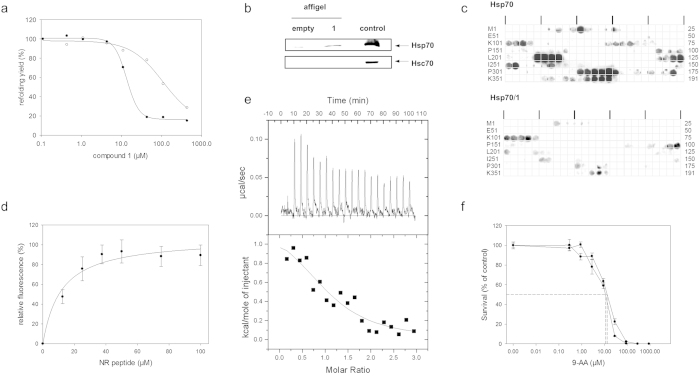
Characterization of the Hsp70 inhibition by 1. (**a**) Inhibitory effect of **1** on the reactivation of GdmCl-denatured firefly luciferase by Hsp70. Refolding was initiated by dilution of 2.08 μM unfolded luciferase into refolding buffer containing Hsp70 (closed circles) or Hsc70 (open circles). The drawn line was calculated using an IC_50_ of 45 μM for Hsp70 and 363 μM for Hsc70. Each data point represents the average of duplicate determinations which differed not more than 10%. (**b**) Derivative **1** interacted with Hsp70 but not with Hsc70 in HeLa cell lysate. Affigel-10 beads pre-incubated with **1** were incubated with HeLa cell lysate. Bound proteins were analyzed by SDS-PAGE and immunoblotting using anti-Hsp70 and anti-Hsc70 antibodies, respectively. As controls, a loading control and affigel-10 beads not pre-incubated with **1** were used. (**c**) Binding of Hsp70 to p53 derived peptides was inhibited by **1**. Binding was detected by incubating a cellulose-bound peptide array of p53-derived 12mer peptides with Hsp70 alone (upper panel) and inhibition of binding was observed in the additional presence of **1** (lower panel). (**d**) The peptide NRLLLTG competed with compound **1** for binding to Hsp70. Analysis was performed in triplicate by a fluorescence-based competition experiment. The continuous lines represent the best fits of the data to a single binding site model. Error bars represent SD. (**e**) Isothermal titration of Hsp70 with **1.** Upper panel: raw titration data; lower panel: integrated enthalpies. A solution of 20 μM Hsp70 (initial concentration) was titrated at 20 °C with 200 μM **1**and fitted to a single-site binding model, resulting in a K_D_ of 5.6 ± 3.1 μM, with stoichiometry N = 1.1, ΔH_ITC_ = 1.3 ± 0.3 kcal mol^−1^, and TΔS_ITC_ = 8.3 kcal mol^−1^. (**f**) The effect of acridizinium derivatives on cell survival using the clonogenic assay. EC_50_ were determined to be 11.2 μM for **1** and 13.1 μM for **3**.

Compound **1** potently inhibited the folding helper activity of Hsp70 with an IC_50_ value of 45 ± 4 μM, as determined by the dose dependent decrease of the yield of native protein from the GdmCl-denatured firefly luciferase in a refolding assay assisted by Hsp70 (Table 1, Fig. 1a). The ATPase activity of Hsp70 remained unaffected at concentrations up to 200 μM of **1** (Supplementary information, Fig. S1). To evaluate the selectivity of the inhibitor with respect to different isoforms of the Hsp70 family of chaperones, **1** was additionally analyzed for inhibition of the Hsc70-assisted refolding of firefly luciferase. Compound **1** exhibited a considerable specificity towards Hsp70. Inhibition of Hsc70 was determined to be 8-fold less efficient when compared to Hsp70.

In contrast, the alkylamino-substituted acridizinium derivative **2** which inhibited Hsp70 with an IC_50_ value of 127 ± 7 μM, inhibited Hsc70 with a 10-fold increased affinity compared to Hsp70 (Table 1). The sulfanyl-substituted derivative **3** was also included for comparison, which differs from **1** only in the substituent at position 9 of the acridizinium ring, but that did not significantly inhibit the protein folding helper activity of Hsp70. Thus, derivative **3** can be used as an Hsp70-inert control. Thus, cellular effects arising only by application of **1** but not by application of **3** are probably mediated by Hsp70 inhibition, whereas biological responses towards both compounds are probably due to side effects unconnected with Hsp70 inhibition.

To verify whether **1** is able to bind to endogenous Hsp70, attempts were made to directly analyze the cellular targets of **1** in cell lysate. HeLa cell lysates were incubated with **1** coupled to affigel-10 beads. After extensive washing, complexes of **1** with cellular proteins were analyzed by immunoblotting using specific anti-Hsp70 and anti-Hsc70 antibodies that show no cross-reactivities. Evidence for a Hsp70/(**1**) interaction specific amongst the Hsp70 family in the cell was established by detection of Hsp70 but no detectable amount of Hsc70 bound to matrix-coupled **1** (Fig. 1b).

### Binding of **1** to the Hsp70 peptide binding site

We next investigated if recombinant Hsp70 binds **1** via its C-terminal peptide binding site which is identical to the APIase catalytic cleft in the Hsp70 chaperone DnaK^14^. This protein region constitutes a channel defined by loops made up from the beta sandwich structure of the C-terminal substrate-binding domain^41^. A novel competition assay was established by designing an overlapping library of matrix bound 12mer oligopeptides of the known Hsp70 binding protein p53^42^. Hsp70 binding on the matrix-bound p53 peptide library was used to reveal whether Hsp70 suffers the loss of affinity in the presence of **1** (Fig. 1c). The p53 derived oligopeptide scan revealed six major Hsp70 binding regions encompassing the sequence stretches ^23^WKLLPENN^30^, ^107^YGFRLGF^113^, ^215^SVVVPYE^221^, ^251^ILTIITL^257^, ^329^TLQIRGR^335^ and ^376^STSRHKK^382^. In the presence of **1** Hsp70 binding to five of the six interacting regions was abolished. This result indicated the ability of **1** to displace the 12-mer peptides from the Hsp70/peptide complex by competitive binding to the same site of Hsp70 (Fig. 1c). Only Hsp70 binding to peptides of the p53 region ^107^YGFRLGF^113^ remained unaffected indicating an alternative binding site for peptides encompassing this sequence region independent of **1**.

Additionally we analyzed the binding of **1** to Hsp70 by a fluorescence-based competition assay^43^. Increasing amounts of the peptide NRLLLTG (NR peptide) were administered to a solution of Hsp70 bound to **1**. The NR peptide was shown to bind to the peptide binding site of Hsp70^39^,^43^. The interaction of Hsp70 and **1** resulted in a decrease of fluorescence signal of 1. Increase of fluorescence upon addition of **1** indicated the ability of the NR peptide to displace compound **1** from the Hsp70/**1** complex (Fig. 1d). The competition assay revealed an IC_50_ value of 11.5 ± 3.8 μM for the NR peptide which is similar to the K_D_ of 9 μM already described for the NR peptide^43^.

To determine the thermodynamic parameters of the association reaction between Hsp70 and 1, ITC experiments were performed. The titration of 20 μM Hsp70 with 200 μM **1** was performed at 20 °C and pH 7.8 (Fig. 1e). It revealed a binding enthalpy ΔH_ITC_ of 1.3 ± 0.3 kcal mol^−1^, a TΔS_ITC_ of 8.3 kcal mol^−1^ and a ΔG_ITC_ of −7.0 kcal mol^−1^ which results in a dissociation constant K_D_ of 5.6 ± 3.1 μM. The positive ΔH and the positive TΔS showed that the association of Hsp70 with **1** was enthalpically disfavoured but entropically driven. The positive entropic contribution hints to a burial of solvent-accessible surface area on binding, since the release of ordered water molecules to bulk solvent often contributes to a favorable entropic contribution to the interaction.

### Cytotoxicity of the Hsp70 inhibitor

In order to examine the influence of Hsp70 inhibition in living mammalian cells, cytotoxicity associated with the application of acridizinium derivatives was determined.

First, various human cell lines including cervix (HeLa), kidney (HEK-293), colon (HT-29) and neuroblastoma (SH-SY5Y) cells were analyzed by means of the MTT assay for a cytotoxic action of **1** and **3** (Table 2). Both compounds inhibited cell survival dependent of the cell line used with EC_50_ values in the similar range, which indicates that the cytotoxicity of the compounds is independent of Hsp70. However, cell viability of Vero cells was not impaired during shorter incubation times i.e. 2–5 h (Supplementary information, Fig. S2).

To evaluate the long-term cytotoxicity of the compounds, we analyzed the effect of **1** and **3** on the survival of HeLa cells in a clonogenic assay (Fig. 1f). HeLa cells were plated after incubation with increasing concentrations of both compounds for 28 h at 37 °C. The colonies formed by surviving HeLa cells were counted after **2** weeks incubation, and survival was calculated as compared with control cells. The obtained dose-response curve allowed the estimation of IC_50_ values of 11.2 μM for **1** and 13.1 μM for **3** (Fig. 1f). Shorter exposure to the compounds resulted in a decreased sensitivity of cells. Similar cellular sensitivity against **1** and **3** indicates that Hsp70 inhibition does not contribute to long-term cytotoxicity. HPLC analyses of the lysates after 24 h incubation of the cells have provided evidence that the compounds are not only cell permeable but also chemically stable under these conditions (Supplementary information, Fig. S3).

Taken together, our results show some cytotoxic effects of the acridizinium compounds independent of Hsp70 inhibition. Cytotoxic effects occurred after comparatively long incubation periods of 28 resp. 48 h under the respective experimental conditions. The similar ability of compounds **1** and **3** to bind to DNA^44^,^45^,^46^, suggests cytotoxicity related to the DNA-binding properties of the compounds. However, these cytotoxic effects limit the application of the compounds for intracellular Hsp70 inhibition to concentrations in the lower micromolar range and incubation times not longer than 24 h.

### Influence of **1** on Hsp70 localization after heat shock

We next examined the ability of acridizinium compounds on cell morphology and the subcellular distribution of Hsp70 in HeLa cells under both, normal and heat stress conditions.

**Figure 2:**
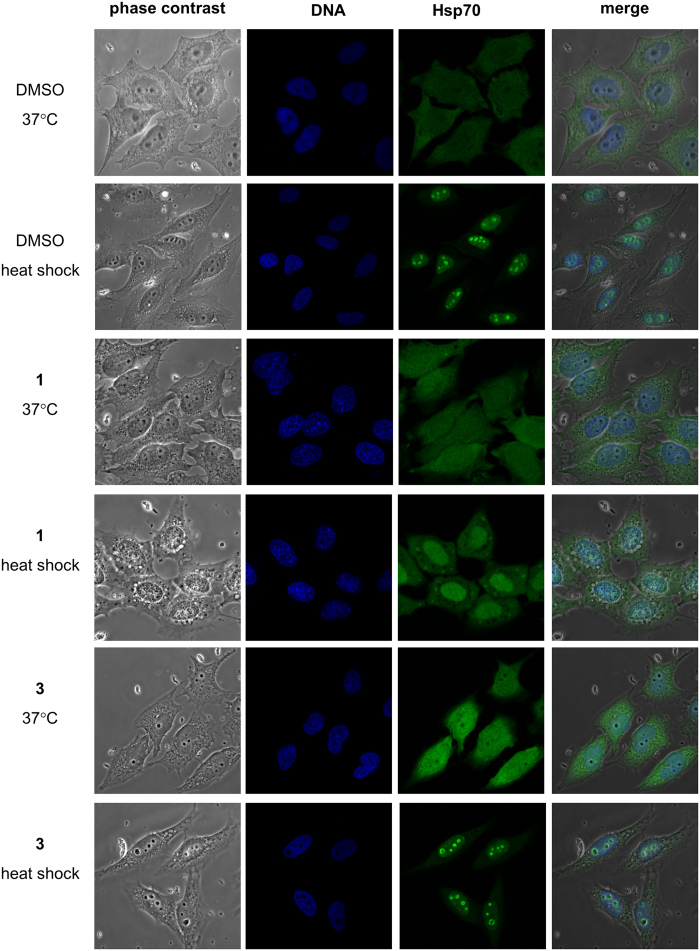
Hsp70 inhibition changed localization of Hsp70 in HeLa cells after heat shock. Hsp70 localized throughout HeLa cells 37 °C localized to nucleoli after heat shock for 1 h at 42 °C. Exposure of the cells to the acridizinium derivative **1** inhibited Hsp70 relocalization after heat shock whereas the inactive acridizinium derivative **3** did not influence Hsp70 relocalization to the nucleoli after heat shock. Cells were fixed and labeled with antibodies to Hsp70 (green). DNA was stained by DAPI (blue).

We found that the presence of **1** induced a strong cytoplasmic vacuolization of the HeLa cells after heat stress as identified by phase contrast microscopy (Fig. 2). Neither HeLa cells in the absence of **1** nor in the presence of **3** showed a comparable formation of vacuoles.

It is well known that Hsp70 alters its localization upon heat shock. The cytoplasmic distribution of Hsp70 observed at 37 °C is changed resulting in nucleolar localization at 42 °C (Fig. 2). The presence of **1** did not alter the localization of Hsp70 at 37 °C. However, **1** caused a dramatic Hsp70 re-distribution into the whole nucleus at 42 °C whereupon the nucleolar accumulation disappeared. The control compound **3** did not alter the cellular distribution neither at 37 °C nor at 42 °C.

In conclusion, the presence of **1** prohibits the accumulation of Hsp70 in the nucleolus during heat stress. This suggests that **1** inhibits Hsp70 targeting of its substrate proteins that accumulate in the nucleolus under heat stress conditions.

### Induction of apoptosis and cell cycle arrest

**Figure 3:**
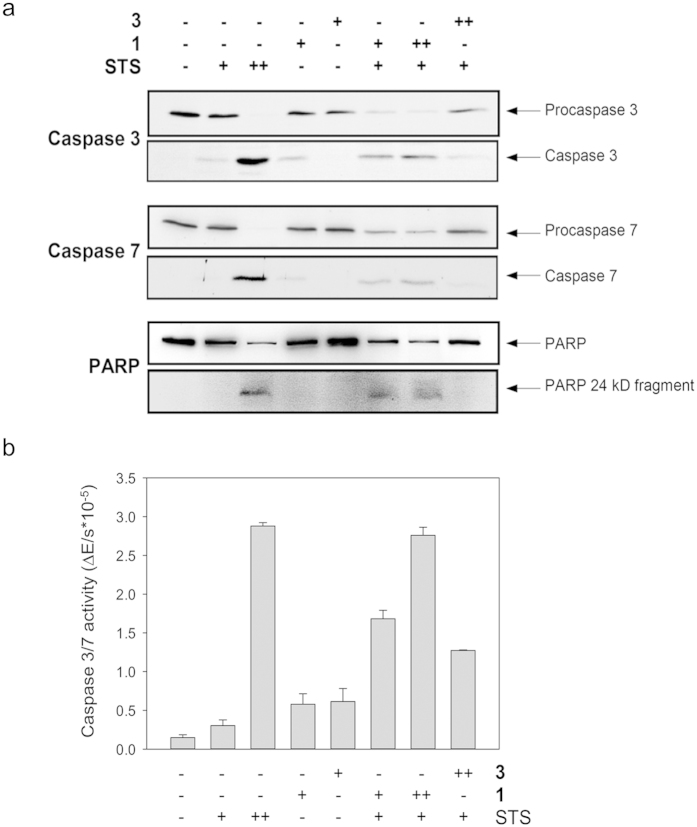
Synergistic activation of apoptosis by staurosporine and 1. Induction of apoptosis in HeLa cells by **1** required a co-stimulus such as staurosporine. (**a**) Analysis of the proteolytic cleavage caspase-3 proenzyme, caspase-7 proenzyme, and of poly (ADP-ribose) polymerase (PARP). Confluent HeLa cells were exposed to **1**, **3** or staurosporine or a combination of the acridizinium derivative and staurosporine. The combination of **1** and staurosporine increased the proteolytic cleavage indicative of apoptosis. Proteins of the cell lysates were resolved by SDS-PAGE and analyzed by immunoblotting. (**b**) Activation of caspase 3/7 during apoptosis in the presence of acridizinium derivatives and staurosporine. Cytosolic extracts were incubated with the caspase 3/7 specific substrate Ac-DEVD-pNA and absorbance of p-nitroaniline proteolytically released by active caspase 3/7 was measured at 405 nm. Staurosporine was applied at 0.1 μM (+) or 0.4 μM (++), the concentration of **1** was 177 μM (+) or 355 μM (++) and the concentration of **3** was 320 μM (++).The combination of **1** and staurosporine increased the proteolysis indicating active caspase 3/7.

Because of the known involvement of Hsp70 in apoptosis, we analyzed the influence of Hsp70 inhibition on apoptosis of HeLa cells. The profiles of caspase 3 and 7 activation were established by Western blot analysis (Fig. 3a). The administration of 0.4 μM staurosporine was found to result in the cleavage of both procaspases, whereas 0.1 μM staurosporine did not result in significant cleavage. However, combined administration of 0.1 μM staurosporine and micromolar concentrations of **1** induced a synergistic effect in that cleavage of both, procaspases 3 and 7 and concomitant formation of the active caspase fragments was observed.

Even in concentrations as low as 10 μM **1** enhanced apoptosis induction by staurosporine (Supplementary information, Fig. S4a). In addition, the activities of caspases 3 and 7 were measured by the use of a colorimetric assay and showed results similar to those obtained by Western blotting (Fig. 3b). Application of the staurosporine/**3** combination or **1** or **3** alone did not result in significant procaspase cleavage. Noteworthy, also the presence of the counterions BF_4_^−^ or Br^−^ in combination with staurosporine had no significant effect on the formation of active caspase fragments (Supplementary information, Fig. S4b). Among the endogenous proteolytic substrates specifically cleaved during apoptosis is poly (ADP-ribose) polymerase (PARP)^47^. Cleavage of the 116 kDa PARP with the concomitant formation of the PARP cleavage product of 24 kDa was induced by the combined administration of staurosporine and **1**, whereas the compound alone applied at the same concentrations had no effect. These results show that application of **1** sensitized cells to an apoptotic insult whereas it did not induce apoptosis on its own. Moreover, the finding that compound **1** modulates a well-known cellular function of Hsp70, i.e. the anti-apoptotic effect, served as a proof-of-concept of the functionality of compound **1** in living cells.

**Figure 4:**
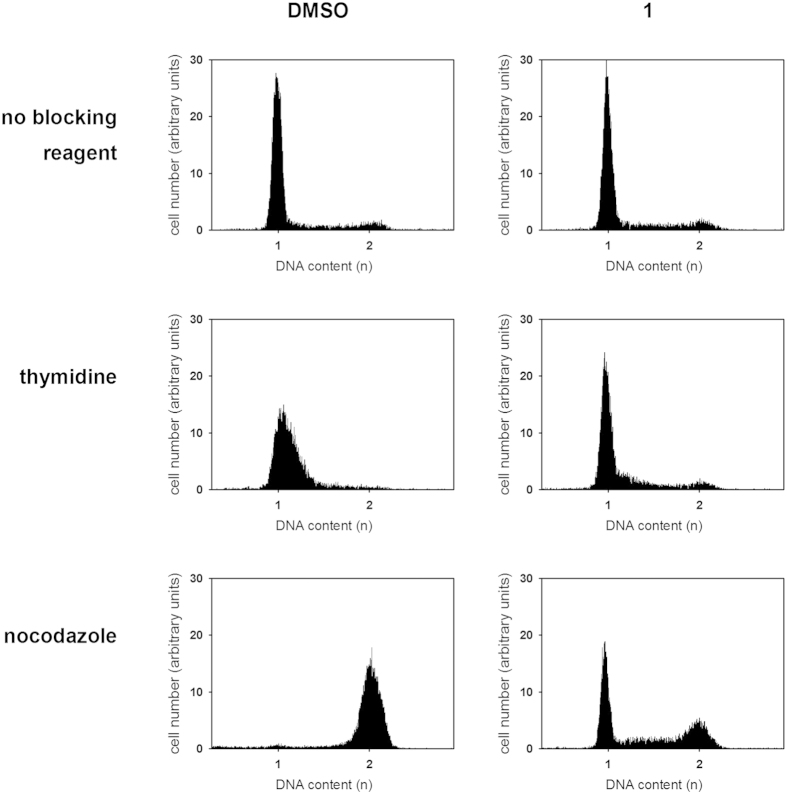
Hsp70 inhibition changes cell cycle distribution of HeLa cells. The application of **1** induces G1 arrest in HeLa cells. HeLa cells were preincubated for 1 h at 37 °C in presence of 0.4% DMSO or 25 μM **1**. Following this, cells were treated with thymidine, nocodazole or diluent for 20 h before the harvest. The HeLa cells were analyzed for the DNA content by flow cytometry. The positions of the G1 (1) and G2/M (2) peaks are marked. The experiment was performed three times with the same results.

We next evaluated the cell cycle effect of **1** on cultured HeLa cells using flow cytometric analysis. Exponentially growing HeLa cells were exposed to 25 μM of **1** for 21 h. This treatment did not result in changes in cell cycle phase distribution (Fig. 4). Repeating the experiment with an imposed cell cycle arrest revealed a considerable impact of Hsp70 inhibition on the cell cycle. Whereas nearly all thymidine-treated control cells accumulated in G1/S, >20% of the additionally Hsp70-inhibited cells remained in G2/M. On the contrary, nearly all nocodazole-treated control cells accumulated in G2/M, whereas the additionally Hsp70-inhibited cells did not accumulate in the G2/M phase but rather showed a phase distribution resembling untreated cells. Analysis of the subcellular distribution of Hsp70 of thymidine-treated and nocodazole-treated cells showed, that the additional presence of **1** did not change the diffuse distribution of the protein in the nucleus and the cytoplasm (Supplementary information, Fig. S5). Notably, application of compound **1** did not influence the amount of Hsp70 in HeLa cells (Supplementary information, Fig. S6).

Taken together, **1** was found to perturb cell cycle phase arrest although the compound by itself did not affect the cell cycle profile.

### Pharmacological inhibition of Hsp70 in cells prevents transport of the enzyme component of internalized *C. perfringens* iota toxin across membranes and thus protects cells from intoxication

After demonstrating Hsp70-related specificity, inhibitory efficacy, tolerability in mammalian host cells and the ability to alter Hsp70 functions in the cell, experiments in the presence of acridizinium compounds to investigate whether Hsp70 is involved in the translocation of Ia, the enzyme component of the *C. perfringens* iota toxin, into host cells became possible.

Since the enzyme components of clostridial binary actin ADP-ribosylating toxins interact with Hsp90 and PPIases *in vitro* and in living cells^18^,^19^, we first analyzed, if these toxins physically interact with Hsp70 and/or Hsc70. Therefore, dot blot analysis was performed to test whether the enzyme components from *C. botulinum* C2 toxin (C2I) and from iota toxin (Ia) directly bind to Hsp70 *in vitro*. Purified Hsp70 and Hsc70 were spotted onto a nitrocellulose membrane followed by overlay with biotin-labeled C2I or Ia.

**Figure 5:**
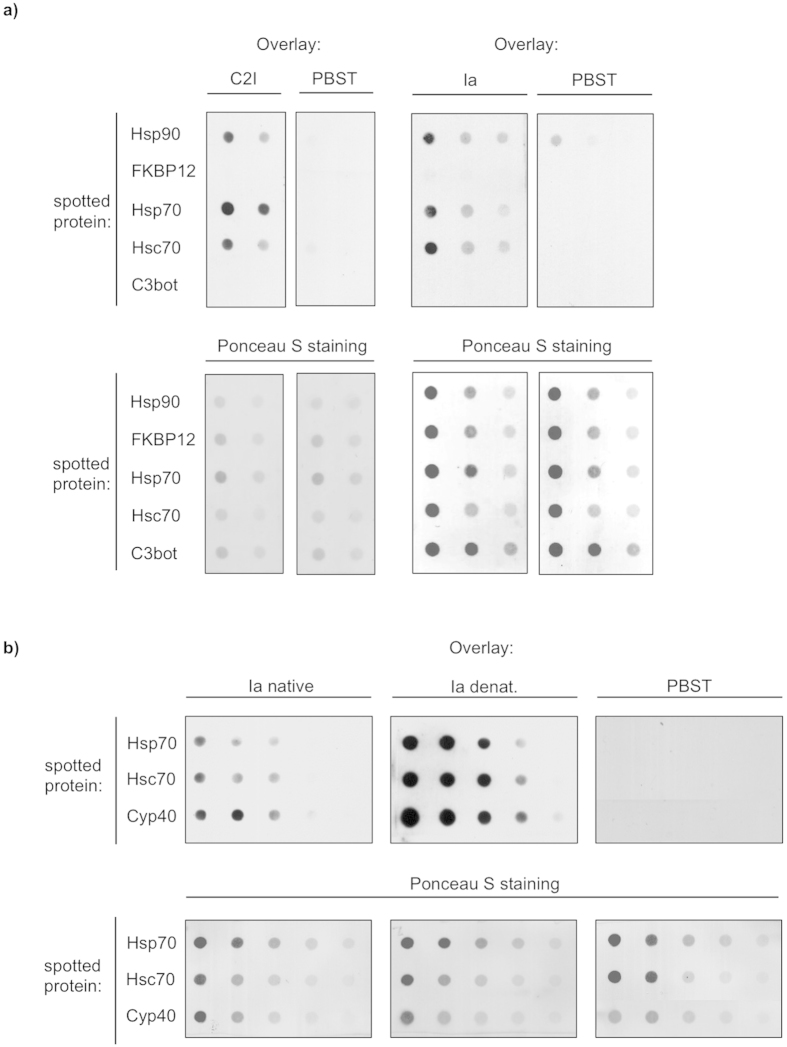
The enzyme components C2I of *Clostridium (C.) botulinum* C2 toxin and Ia of *C. perfringens* iota toxin directly interact with Hsp70 and Hsc70. (**a**) Decreasing amounts of purified proteins Hsp70, Hsc70, Hsp90 (for positive control) and FKBP12 and C3bot (for negative control) were vacuum-aspirated onto a nitrocellulose membrane by using the dot blot system. The membrane was blocked with PBS-T containing 5% nonfat dry milk and incubated with biotin-labeled C2I, Ia (200 ng ml^−1^) or with PBS-T for control for 1h. After washing, the bound enzyme components were detected by streptavidin-POD using the ECL system (upper panel). Successful immobilization of purified proteins was confirmed by Ponceau S staining (lower panel). (**b**) The denatured enzyme component Ia shows stronger binding to Hsp70 and Hsc70 compared to the native form. The experiment was performed as described in a) except that one portion of Ia was incubated in 6 M guanidinium hydrochloride (denatured Ia) prior to overlay while another portion of Ia was incubated in PBST (native Ia).

The result shown in Fig. 5 revealed direct binding of Hsp70 or Hsc70 to C2I and Ia. These interactions were specific since the enzyme components did not bind to the non-relevant C3bot protein or to the membrane and there was no signal when buffer was applied instead of biotin-C2I or biotin-Ia (Fig. 5). Interestingly, the PPIase FKBP12 did not bind C2I or Ia.

Furthermore, we investigated the interaction of Hsp70 and Hsc70 with the denatured enzyme component Ia of the iota toxin. Denaturation was performed by incubation of Ia in 6 M guanidinium hydrochloride. The denatured enzyme component showed a significantly stronger binding compared to the native form to Hsp70 and Hsc70 as well as to the control Cyp40, which was demonstrated earlier^24^. Noteworthy, the binding of denatured Ia was still specific since there was no signal detectable for the interaction with C3bot, which served as a negative control (not shown).

**Figure 6:**
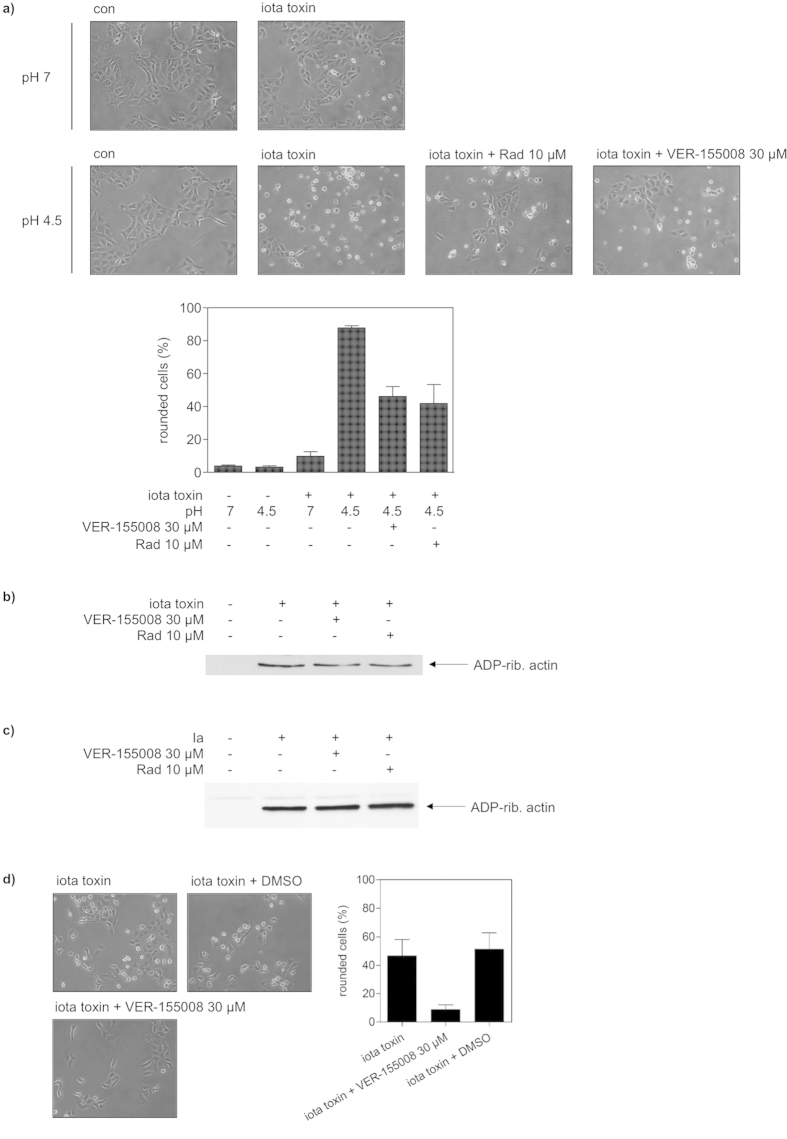
Pharmacological inhibition of Hsp/Hsc70 activity inhibits membrane translocation of the enzyme component Ia of *Clostridium (C.) perfringens* iota toxin but has no effect on receptor binding and enzyme activity of the toxin. (**a**) For the toxin translocation assay, Vero cells were pre-incubated with VER-155008 or radicicol (Rad) in combination with bafilomycin A1 (BafA1) and for control with BafA1 alone for 30 min at 37 °C. After binding of iota toxin (200 ng ml^−1^ Ia plus 200 ng ml^−1^ Ib) at 4 °C, cells were challenged with warm acidic medium to allow the direct translocation of Ia across the cytoplasmic membrane into the host cell cytosol. For control, cells were incubated with neutral medium. Subsequently, cells were further incubated at 37 °C and cell morphology was monitored. The percentage of rounded cells was determined from images taken after 2.75 h of incubation. Values are given as mean ± SD (n = 3). (**b**) Inhibition of Hsp70 activity has no effect on receptor binding of iota toxin. After pre-incubation with VER-155008 or Rad (for control cells were left untreated), Vero cells were cooled to 4 °C and iota toxin was added for 30 min (200 ng ml^−1^ Ia plus 200 ng ml^−1^ Ib). After washing, the cell-bound iota toxin was detected by analyzing the ADP-ribosyltransferase activity of the cell-associated Ia *in vitro* by Western blotting. (**c**) Inhibition of Hsp70 does not interfere with enzyme activity of Ia *in vitro*. (**d**) The Hsc/Hsp70 inhibitor VER-155008 inhibits intoxication of Vero cells with *C. perfringens* iota toxin. Pictures show the toxin-induced morphological changes after 4.5 h of toxin treatment.

Prompted by this result, we tested whether Hsp/Hsc70 are functionally involved in the pH-dependent translocation of Ia across cell membranes, as we observed earlier for Hsp90 and the PPIases. To this end, Vero cells were pre-treated with bafilomycin A1 to inhibit the normal uptake of iota toxin. In addition, some cells were treated with the general inhibitor of the ATPase activity of Hsp70s, VER-155008 and some others with the Hsp90 inhibitor radicicol for positive control. The translocation of Ia from cell-bound iota toxin was investigated in the presence and absence of VER-155008 or radicicol by mimicking the pH-driven transport of Ia across endosomal membranes on the cell surface. Iota toxin was bound to cells at 4 °C and exposed to acidic medium to trigger insertion of Ib into the cytoplasmic membrane where it forms trans-membrane pores which serve for translocation of Ia across the cytoplasmic membrane of the cells into the cytosol. Translocated Ia ADP-ribosylates actin and thus results in cell rounding, the specific endpoint to monitor Ia translocation. Only cells treated with iota toxin and exposed to acidic medium rounded up (Fig. 6a). Importantly, after pre-treatment with VER-155008, there were significantly less round cells, comparable to cells pre-treated with radicicol (Fig. 6a), indicating that the ATPase activity of Hsp70s is crucial for the pH-driven translocation of Ia across the cell membrane. Because VER-155008 (as well as radicicol) had no effect on the enzyme activity of Ia (Fig. 6c) or the binding of iota toxin to the cells (Fig. 6b), these results strongly suggest that Hsp70 facilitates the translocation of Ia from acidic endosomes into the cytosol during normal toxin uptake.

**Figure 7:**
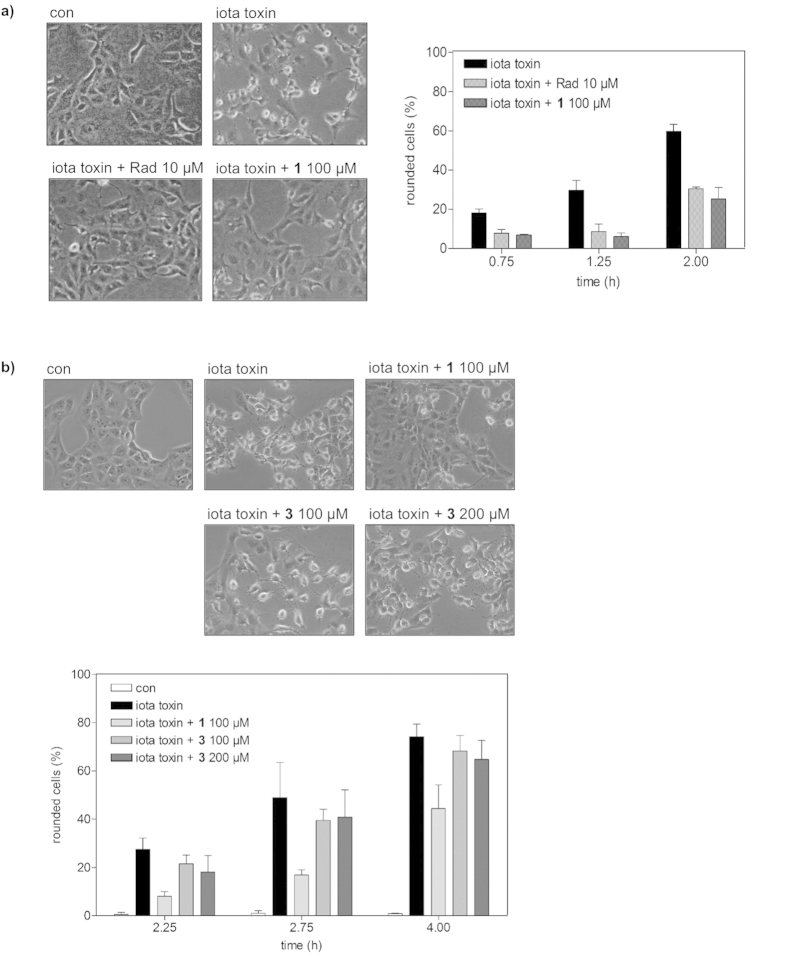
Compound 1 but not 3 inhibits intoxication of Vero cells with *Clostridium (C.) perfringens* iota toxin. (**a**) Influence of **1** on intoxication of Vero cells with *C. perfringens* iota toxin. Vero cells were pre-incubated for 30 min at 37 °C with 100 μM **1** and for control with 10 μM radicicol (Rad) or left untreated for control. Subsequently, iota toxin (50 ng ml^−1^ Ia plus 100 ng ml^−1^ Ib) was added and cells were further incubated at 37 °C. Pictures show the morphological changes after 2 h of toxin treatment. For quantitative analysis, percentage of rounded cells was determined from the pictures taken at the indicated time points. Values are given as mean ± SD (n = 3). (**b**) The inactive analog **3** of the Hsp70 inhibitor **1** has no effect on intoxication of Vero cells with iota toxin. Vero cells were pre-incubated for 30 min at 37 °C with 100 μM **1**, 100 μM **3** or 200 μM **3** or left untreated (control). Subsequently, iota toxin (50 ng ml^−1^ Ia plus 100 ng ml^−1^ Ib) was added and cells were further incubated at 37 °C. Pictures show the morphological changes after 4 h of toxin treatment. For quantitative analysis, percentage of rounded cells was determined from the pictures taken at the indicated time points. Values are given as mean ± SD (n = 3).

Therefore, we investigated the role of Hsp70 during uptake of iota toxin into Vero cells and tested the effects of VER-155008 as well as **1** on the intoxication of cells with iota toxin. Reasoning that a pharmacological blockade provides direct evidence for a functional participation, Vero cells pre-treated with VER-155008 or **1**, were challenged with iota toxin and the toxin-induced cell-rounding was analyzed as endpoint for the uptake of enzymatic active Ia into the cytosol. As shown in Fig. 6d, pre-treatment of cells with VER-155008 had an inhibitory effect on intoxication of the cells with iota toxin. Similar to radicicol, which was used for positive control, pre-treatment of cells with 100 μM **1** significantly delayed the toxin-induced cell-rounding at all of the time points investigated (Fig. 7a). Under the experimental conditions used in this study, there is no evidence that compound **1** might block the Ib pores in the membranes of Vero cells (not shown). Importantly, the inactive analog **3** of the Hsp70 inhibitor **1** had no comparable inhibitory effect on the intoxication of Vero cells with iota toxin at concentrations up to 200 μM (Fig. 7b). Moreover, application of the NR peptide, which binds to the peptide binding site of Hsp70 and prevents interaction of Hsp70 with client proteins, into the culture medium had no effect on the intoxication of Vero cells with iota toxin (not shown), indicating that extracellular Hsp70 plays no role for the uptake of iota toxin.

Taken together, we identified and characterized a novel compound, which acts as specific Hsp70 inhibitor in living mammalian cells. By using this inhibitor, we were able to identify Hsp70 as a novel functional interaction partner of the enzyme component of the binary actin ADP-ribosylating *C. perfingens* iota toxin. Moreover, we demonstrated for the first time that Hsp70 activity of mammalian host cells is crucial for the cellular uptake of a bacterial protein toxin because Hsp70 facilitates the delivery of the enzyme component of the internalized toxin across cell membranes into the cytosol.

## Discussion

In the cell, Hsp70 chaperones are often found to cooperate with Hsp90, a molecular chaperone of particular importance to protein homoeostasis. Together with co-chaperones and PPIases they form multichaperone complexes important for the activation, folding, subcellular localization, and maturation of their client proteins. We reported earlier that Hsp90 and members of two families of PPIases, namely cyclophilins and FK506 binding proteins (FKBPs) facilitate the intracellular membrane transport of the enzyme components of internalized clostridial binary actin ADP-ribosylating toxins from acidic endosomes into the host cell cytosol. Therefore the question arose, whether the process of toxin translocation involves the complete machinery for assisted protein folding including Hsp70 chaperones.

To evaluate the effects of Hsp70 in toxin translocation in living mammalian cells, we searched for a small molecule inhibitor of Hsp70 directed to its peptide binding site. Since we already demonstrated the identity of the peptide binding site and the APIase site for the bacterial Hsp70 homolog DnaK^14^,^30^ an assay based on the catalyzed reactivation of denatured luciferase^48^ in the presence of recombinant human Hsp70 was used. Based on this assay, the acridizinium derivative **1** with a molecular mass of 282 Da was identified from a small in-house library of compounds as a specific Hsp70 inhibitor exhibiting an IC_50_ value of 45 ± 4 μM. ITC measurements revealed a K_D_ value of 5.6 ± 3.1 μM for the Hsp70/1 complex. The compound did not influence the ATPase activity of Hsp70 but abolished Hsp70 binding to p53-derived peptides known to have affinity to the Hsp70 peptide binding site^42^ and directly competes with the NR peptide for binding to Hsp70 (Fig. 1d). Therefore it can be assumed, that docking of the acridizinium derivative concomitantly blocks the peptide binding site of Hsp70.

For analyzing whether **1** is able to modulate a known cellular function of Hsp70, we examined its effect in programmed cell death. Application of the Hsp70 inhibitor alone to unstressed cells had no significant influence on apoptosis. However, consistent with the anti-apoptotic functions of Hsp70 we could show that **1** rendered staurosporine-treated cells more susceptible to apoptosis induction. This effect was judged by the activation and proform cleavage of the effector caspases 3 and 7.

Notably, Hsp70 is a well-known negative regulator of apoptosis that interferes with the action of a variety of apoptotic key proteins. Thus, Hsp70 has been found to interact with the apoptosis protease activating factor-1 thereby inhibiting activation of the caspase cascade^49^. Overexpression of Hsp70 inhibits apoptosis and prevents caspase activation upon a variety of cellular stresses, including the accumulation of misfolded proteins, the presence of reactive oxygen species or DNA damage^50^,^51^. Consistent with our results, depletion of Hsp70 by antisense constructions or application of siRNA increased the sensitivity of cells to apoptotic stimuli^52^,^53^. The Hsp70 binding peptide ADD70 derived from the apoptosis inducing factor AIF directed toward the peptide binding domain sensitized human cancer cells to the chemotherapeutic agents staurosporine and cisplatin^54^. Also the ATPase-directed Hsp70 and Hsc70 inhibitor VER-155008 sensitized colon HCT116 carcinoma cells to die in combination with other drugs^34^. Taken together this suggests that disabling Hsp70 by inhibition of either peptide binding or ATPase activity or by depletion of the protein results in the reduction of cytoprotection and thus in lowering the efficacy threshold against apoptotic stimuli. In contrast to **1**, PES that interacts with the C-terminal part of Hsp70^39^ was shown to induce the apoptotic caspase-dependent cell death independent of external stimuli^38^. These results point toward differences in the biochemical mode of action of **1** and PES as the latter compound does not exhibit affinity to the canonical peptide binding site of Hsp70^39^,^40^. Thus, the approach outlined here allowed the identification of acridizinium derivatives as the first low molecular weight compounds directed to the peptide binding site of Hsp70.

To further characterize the effects of **1** on Hsp70 functions in living cells, we investigated the implications of administering **1** for the intracellular localization of Hsp70. Hsp70 is known to show diffuse cytoplasmic and nuclear localization at physiological temperature in asynchronous populations of cells^55^. Upon heat stress, it was shown to migrate to the nucleus and associate with the nucleoli. For the nucleolar localization, the substrate binding domain, was shown to play a crucial role^55^. Hsp70 is considered to accumulate in the nucleoli along with denatured substrates to assist folding during the recovery of the stressed cells^56^. Interestingly, **1** abolished the association of Hsp70 with the nucleoli. This suggests suppression of formation of Hsp70 complexes with denatured substrate proteins by **1** given these complexes form a prerequisite for nucleolar localization of Hsp70^56^. Notably, **1** induced cell vacuolization under heat stress conditions. Similarly, PES was shown to induce cytoplasmic vacuolization^38^. Investigations on vacuolization induced by Hsp90 inhibition in combination with proteasome blockade lead to the suggestion, that stressors promoting the accumulation of misfolded proteins may have the potential to induce cell vacuolization^57^. Because Hsp70 is translocated to the nucleus and the nucleoli not only under stress conditions but also during the S-phase^55^, we analyzed the impact of **1** on the cell cycle. Despite being ineffective for Hsp70 redistribution, **1** prevented the thymidine-induced accumulation of cells in the S phase as well as the nocodazol-caused arrest of the cells in the G2/M phase of the cell cycle.

Having established that **1** serves as a specific pharmacological inhibitor for Hsp70 in living cells, we exploited **1** to investigate whether in addition to Hsp90 and PPIases, Hsp70 plays a role for the uptake of bacterial ADP-ribosylating protein toxins into mammalian cells. To this end, we pre-treated cells with the Hsp70 inhibitor **1** prior to application of the *C. perfringens* iota toxin and monitored the toxin-induced cell rounding in comparison to cells treated with iota toxin in the absence of the inhibitor. Similar as after application of radicicol, a specific inhibitor of Hsp90, the formation of rounded cells indicative of intoxication was strongly reduced, implicating that less of the enzymatic active component Ia reached the host cell cytosol in the presence of inhibitor **1**. Noteworthy, **1** had no cytotoxic effect on Vero cells under the respective experimental conditions. The comparison of the effects of the Hsp70 targeted **1** and the Hsp70 inert **3** in living mammalian cells allowed to discriminate between the alternative reaction modes of the compounds. In general, DNA-binding properties have been described for different quinolizinium derivatives^58^. Specifically, acridizinium derivatives **1** and **3** were shown to intercalate into duplex DNA, which may give rise to genotoxic effects. However, since the ability to bind to DNA is very similar for both, compounds **1** and **3**^44^,^45^,^46^, the different effects of **1** and **3** in living cells imply an Hsp70 related mode of action in inhibition of intoxication and exclude non-specific effects such as elevation of endosomal pH. Thus, the inhibition of Hsp70 directly indicated the importance of an unperturbed peptide binding site for the presence of the functional toxin in the cytosol of target cells.

Dot blot experiments implicated the enzyme components of C2 and iota toxins as interaction partners of both Hsp70 and Hsc70 *in vitro*. We confirmed in our earlier studies that only ADP-ribosylating toxins and their isolated ADP-ribosyltransferase domains bind to the purified chaperones/PPIases in the dot blot assay, but not the transport subunits (e.g. C2IIa^24^) enzyme subunits of bacterial protein toxins which are not ADP-ribosyltransferases such as the anthrax lethal factor^59^. Moreover, the denatured form of the enzyme component of the iota toxin showed a stronger binding compared to the native form. This result is plausible since the enzyme component has to be at least partially unfolded to translocate through the narrow pore formed by the binding/translocation component into the host cell cytosol. Hsp70 is also known to interact with unfolded proteins and probably interacts with the translocating i.e. unfolded enzyme component in the cell. The enhanced binding of enzyme components in their unfolded conformation was also observed for other host cell factors such as FKBP51 and Cyp40^23^,^24^. It is believed that the peptide binding domain in isolation carries the function of the full-length Hsp70^36^,^37^. However, Hsp70 action during toxin translocation required both a functional peptide binding site and a working ATPase site. Consequently, inhibition of the ATPase activity of the Hsp70s by VER-155008 showed an inhibitory effect on intoxication with iota toxin similar to **1**. We reported earlier that inhibition of Hsp90 prevented the pH-dependent transport of the enzyme components of iota toxin and related binary toxins across endosomal membranes and thereby prevented their translocation from acidified endosomal vesicles into the cytosol, but had no effects on the earlier steps of toxin uptake or on the enzyme activities of the toxins^18^,^19^. Here, we demonstrated for the first time that the targeted pharmacological inhibition of Hsp70 prevented the pH-dependent translocation of the enzyme subunit of an internalized bacterial protein toxin, namely *C. perfringens* iota toxin, across cell membranes, which implicates a role for Hsp70 in translocation of this toxin from the lumen of acidified endosomes into the host cell cytosol. This result also excludes the elevation of endosomal pH as a potential reason for the inhibited cell intoxication. However, pharmacological inhibition of Hsc/Hsp70 did not interfere with the enzyme activity of the toxin or binding of the toxin to the cell surface receptors.

Moreover, pharmacological inhibition of Hsp70 also protected mammalian cells from intoxication with further binary clostridial actin ADP-ribosylating toxins including *C. botulinum* C2 toxin and *C. difficile* CDT (unpublished results), suggesting a common Hsp70-dependent membrane translocation mechanism for this toxin family^28^.

In conclusion, we identified and characterized a novel specific Hsp70 small molecule inhibitor that selectively inhibits the protein binding site of Hsp70 providing the possibility to investigate novel cellular functions of Hsp70 in living cells, for example in the context of intoxication with bacterial toxins. To our knowledge, the results of the present study are the first observation that host cell Hsp70 is crucial for the cytotoxic mode of action of a bacterial protein toxin by facilitating the intracellular membrane transport of its enzymatic active component into the cytosol of mammalian target cells.

## Methods

The recombinant C2I protein was purified and activated as described before^60^. Recombinant C3bot was purified as described earlier^61^. Ia and Ib were prepared as described earlier^62^. Recombinant Hsp70, Hsc70 and Hsp90 proteins were purified as described^63^,^64^. Acridizinium derivatives **1–3** were prepared according to published protocols^65^. Characterization of the compounds including NMR data can be found in the literature^44^,^46^,^65^. VER-155008 was purchased from Sigma-Aldrich.

### Luciferase refolding assay

Refolding experiments were done as described by Szabo *et al.*^66^. Briefly, luciferase from *Photinus pyralis* (Promega) was incubated for one hour in denaturation buffer (30 mM Tris/HCl, pH 7.4, 5 mM DTT, 6.0 M GdmCl) at 10 °C at a final concentration of 2.08 μM. Refolding was initiated by dilution of denatured luciferase into renaturation buffer (10 mM MOPS, pH 7.8, 50 mM KCl, 1 mM ATP, 5 mM MgCl_2_, 1 μM BSA, 1.5 μM Hsp70, 160 nM DnaJ) to a final concentration of 10.4 nm at 10 °C. After one hour of refolding luciferase activity was determined with a luminometer (Luminoskan Ascent) using luciferase assay buffer (20 mM Tricine, pH 7.8, 5 mM MgCl_2_, 0.1 mM EDTA, 3.3 mM DTT, 270 μM coenzyme A, 500 μM D-luciferin, 500 μM ATP). The IC_50_ values have been calculated for a one site competition model with SPSS Sigmaplot 8.0.

Hsp70-assisted luciferase refolding was used for screening an in-house library of 3300 natural compounds in search for inhibitors of Hsp70. Compounds were dissolved in DMSO and used at a concentration of 5 μg/ml. The compounds were incubated in renaturation buffer for 10 min before the refolding reaction was started.

### ATPase activity determination

Different concentrations of **1** were incubated with 0.5 μM Hsp70 for 60 minutes at 37 °C in 40 mM HEPES buffer pH 7.6, 50 mM KCl, 11 mM MgCl_2_, 1 mM ATP. Produced phosphate was detected according to Bartolommei *et al.* 2013^67^.

### Peptide binding analysis

Binding of Hsp70 to p53-derived peptides was assessed by using a cellulose-bound peptide array. Overlapping 12mer peptides spanning the p53 sequence linked via (β-Ala)_2_ spacer onto cellulose membranes were synthesized automatically by Fmoc chemistry using the Autospot Robot ASP 222 (Gilson)^68^. The peptide array was incubated with 500 nM Hsp70 alone or in the additional presence of 50 μM of **1** in 10 mM Tris pH 7.6, 150 mM NaCl, 0.05% Tween 20 and 5% sucrose for 60 minutes at room temperature. Bound protein was analyzed by western blotting and immunodetection using monoclonal anti-Hsp70 antibody (Biomol). Fluorescence-based competition experiments followed a procedure derived from Aprile *et al.*^43^ employing the competition of the peptide NRLLLTG and compound **1**for binding the peptide binding site of Hsp70. After incubation of Hsp70 (9.4 μM) and **1** (1.25 μM) in 20 mM Tris pH 7.8, 150 mM KCl, 5 mM MgCl_2_ at 20 °C, increasing amounts of the peptide NRLLLTG were added. To analyze fluorescent ligand displacement, the fluorescence intensity was measured at excitation and emission wavelengths of 300 and 568 nM, respectively.

ITC experiments were performed using a VP-ITC (MicroCal). Prior to the experiment, all buffers were filtered through filter membranes with a pore size of 0.2 μm (Whatman) and degassed. Protein solutions were dialyzed against the assay buffer (in 20 mM Tris pH 7.8, 150 mM KCl, 5 mM MgCl_2_). In a typical experiment, 300 μl of a 200 μM solution of **1** was titrated in 15 μl-steps to a 20 μM Hsp70 solution at 20 °C. The instruments stirring speed was set to 310 RPM and the feedback gain mode was set to “high”. Since the signal from the first injection can usually not be used for data analysis only 2 μl were titrated in this step and the data point was omitted. Measured data were analyzed using the “Origin” software (MicroCal).

### Cell culture

HeLa cells, HEK-293 cells and SH-SY5Y cells were cultured in DMEM supplemented with 10% fetal bovine serum. HT-29 cells were cultured in McCoy’s 5A medium supplemented with 10% fetal bovine serum. Vero (African green monkey kidney) cells were cultivated in MEM containing 10% heat-inactivated fetal calf serum (FCS), 1.5 g/l sodium bicarbonate, 1 mM sodium-pyruvate, 2 mM L-glutamine and 0.1 mM non-essential amino acids at 37 °C and 5% CO_2_. Cells were detached using trypsin and reseeded no more than 20 times.

To assay HeLa cell colony formation abilities after Hsp70 inhibitor treatment by a clonogenic assay, 1.3 × 10^4^ HeLa cells per well were seeded to 6-well plates and allowed to adhere for 42 h at 37 °C. Cells were treated with various concentrations (0.08 to 250 μg/ml) of Hsp70 inhibitor for 18 h in triplicate. Following this, cells were detached, depleted and seeded to 85 mM petri dishes. After 14 d of incubation, colonies were fixed by 4% formaldehyde in PBS, stained by 0.25% crystal violet in PBS and counted.

To assay HeLa cell, HEK-293 cell, HT-29 cell and SH-SY5Y cell survival after Hsp70 inhibitor treatment by the MTT assay, 440 cells per well were seeded to 96-well plates and allowed to adhere for 18 h at 37 °C. Cells were treated with various concentrations (0.08 to 250 μg/ml) of Hsp70 inhibitor for 48 h in quadruplicate. Thereafter, MTT to the final concentration of 0.3 mg/ml was added, and cells were incubated for additional 2 h. After removing the medium, 100 μl DMSO and 25 μl 100 mM glycine buffer, pH 10.5 were added. Finally, the absorbance at 570 nM was determined. The absorbance corresponding to untreated control cells was assumed as 100% cell viability.

### Apoptosis

Analysis of apoptosis was done using immunoblot analysis of the proteolytic cleavage of caspase-3 proenzyme, caspase-7 proenzyme, and of PARP. Confluent HeLa cells were exposed to 177 or 355 μM of **1**, 320 μM of **3** or 0.1 or 0.4 μM of staurosporine or a combination of one of the 9-aminoacridizinium derivatives and staurosporine as indicated. After 45 min or 4 h incubation, cells were taken to obtain lysates in lysis buffer (50 mM HEPES pH 7.4, 150 mM NaCl, 50 mM KCl, 10 mM MgCl_2_, 0.4 mM EGTA, 0.5% (v/v) NP-40, 0.1% (v/v) CHAPS, 10% (w/v) Saccharose, 0.5 mM PMSF, 1 mM DTT, 0.5% (w/v) Molybdat).

After 10% SDS-PAGE electrophoresis, proteins were transferred to nitrocellulose membranes. The membranes were incubated with anti caspase 3 mouse monoclonal antibody (3G2, Cell Signaling), anti caspase 7 mouse monoclonal antibody (Stressgen) or anti PARP mouse monoclonal antibody (Ab-3, Calbiochem) followed by appropriate secondary antibodies conjugated with horseradish peroxidase. ECL chemiluminescent cocktail (Amersham) was used to develop the immunoblots.

The caspase 3/7 activity was analyzed by *in vitro* proteolytic cleavage of specific substrates. Confluent HeLa cells (1 × 10^6^ cells) were exposed to 177 or 355 μM of **1**, 320 μM of **3** or 0.1 or 0.4 μM of staurosporine or a combination of one of the acridizinium derivatives and staurosporine as indicated. After 4 h incubation, cells were taken to obtain lysates in lysis buffer 2 (50 mM HEPES pH 7.4, 150 mM NaCl, 50 mM KCl, 10 mM MgCl_2_, 0.4 mM EGTA, 0.5% (v/v) NP-40, 0.1% (v/v) CHAPS, 10% (w/v) Saccharose, 0.5 mM PMSF, 1 mM DTT). 800 μl of cell extracts were placed in reduced plastic cuvettes and incubated for 10 min at 25 °C. Subsequently, the caspase-3/7 substrate Ac-DEVD-pNA was added to give a final concentration of 28 μM. The release of p-nitroaniline was measured by detection of the absorbance at 405 nm for 30 min.

### Cell cycle analysis

Exponentially growing HeLa cells were incubated for 1 h at 37 °C in presence of 0.4% DMSO or, additionally, 6.9 μg/ml (25 μM) **1**. Following this, cells were treated with thymidine (2 mM), nocodazole (1.3 μM) or diluent for 20 h before the harvest. The harvested HeLa cells were fixed with ethanol, stained with propidium iodide and analyzed for the DNA content by flow cytometry using a Becton Dickinson FACSort. The experiment was performed three times with the same results.

### Confocal microscopy

HeLa cells grown in μ-slide 8-well plates (ibidi) were incubated either for 3 h at 37 °C or for heat shock for 2 h at 37 °C and for 1 h at 42 °C. The medium contained 0.4% DMSO or, additionally, 100 μg/ml Hsp70 inhibitor. Subsequently, cells were washed with PBS, fixed in 4% formaldehyde, permeabilized by methanol, saturated with 2% glycine and blocked in 0.4% BSA in PBS. DNA was stained with DAPI, Hsp70 was stained with mouse anti Hsp70 antibody and FITC-conjugated anti mouse antibody. Finally, the cells were visualized with a Nikon Confocal C1 microscope.

### Interaction analysis between toxins and immobilized Hsp70 using the dot blot system

Serial dilutions of the purified proteins proteins Hsp70, Hsc70, Hsp90 (for positive control) and FKBP12 and C3bot (for negative control) were vacuum aspirated onto a nitrocellulose membrane using the Bio-Rad dot blot system according to the manufacturer’s manual. A Ponceau S staining was performed and then the membrane was blocked with 5% non-fat dry milk in PBS containing 0.1% Tween-20 (PBS-T). Subsequently, the membrane was cut and probed for 1 h with biotin-labeled C2I or Ia (200 ng/ml) that have either been pre-incubated for 1 h in 6 M guanidinium hydrochloride for denaturation of the enzyme components or in PBST to retain native conformation. After extensive washing, the bound proteins were detected by streptavidin-peroxidase using the ECL system. Successful immobilization of purified proteins was confirmed by Ponceau S staining.

### Intoxication experiments and analysis of the enzyme activity, cell binding, and membrane transport of *C. perfringens* iota toxin

For cytotoxicity assays, cells were seeded in culture dishes and incubated in MEM plus FCS with the respective toxin. Cells were visualized after the indicated incubation times by using a Zeiss Axiovert 40CFI microscope with a Jenoptik progress C10 CCD camera. The toxin-induced changes in cell morphology (i.e. cell-rounding) were analyzed and percentage of round cells was determined from the images as described earlier^23^.

The toxin translocation assay was performed as described before^69^. In brief, Vero cells were pre-incubated with bafilomycin A1 to prevent uptake of the toxin via the endosomal pathway and the respective inhibitors i.e. VER-155008 or radicicol. Subsequently, cells were cooled to 4 °C and iota toxin was added for 20 min at 4 °C to enable toxin binding. This was followed by an acidic pulse, i.e. adding warm acidic medium, allowing the enzyme component to directly translocate across the cytoplasmic membrane into the host cell cytosol. Cells were further incubated at 37 °C and cell morphology was monitored as described previously.

For analyzing *in vitro* enzyme activity of Ia, 20 μg of Vero cell lysate containing protease inhibitor were pre-incubated with the respective Hsp70 or Hsp90 inhibitors for 30 min at 37 °C. Subsequently, the enzyme component Ia as well as biotin-labeled NAD^+^, the co-substrate for ADP-ribosylation, were added and samples were incubated for 20 min at 37 °C. Next, samples were denatured at 95 °C with Laemmli sample buffer to stop the enzyme reaction and then subjected to SDS-PAGE followed by Western Blot analysis to detect biotin-labeled i.e. ADP-ribosylated actin.

To analyze the amount of iota toxin bound on the surface of Vero cells treated with the respective Hsp70 or Hsp90 inhibitors, cells were pre-incubated with VER-155008 or radicicol for 30 min at 37 °C. Cells were cooled to 4 °C and iota toxin was added for another 30 min at 4 °C to enable binding. After extensive washing, cells were lysed and incubated with biotin-labeled NAD^+^ for 30 min at 37 °C. Subsequently, samples were subjected to SDS-PAGE and ADP-ribosylated actin was analyzed by Western blotting with streptavidin-peroxidase. The amount of ADP-ribosylated actin directly correlates with the amount of cell-bound iota toxin and thus allows detection of cell-bound Ia. Comparable amounts of blotted protein were confirmed by Ponceau S staining of the blot membrane.

## Additional Information

**How to cite this article**: Ernst, K. *et al.* A novel Hsp70 inhibitor prevents cell intoxication with the actin ADP-ribosylating *Clostridium perfringens* iota toxin. *Sci. Rep.* **6**, 20301; doi: 10.1038/srep20301 (2016).

## Supplementary Material

Supplementary Information

## Figures and Tables

**Table 1:**
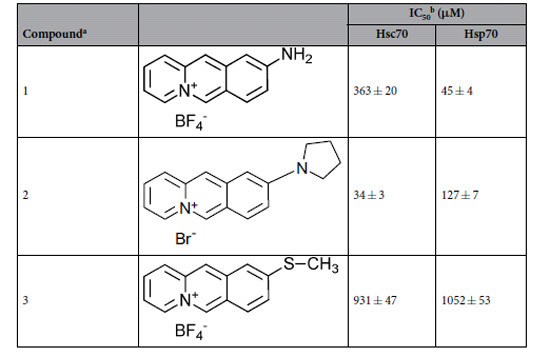
Inhibition of Hsp70 assisted refolding of firefly luciferase by 9-aminoacridizinium derivatives.

^a^Characterization of the compounds including NMR data can be found in the literature^44^,^46^,^65^.

^b^IC_50_ values were determined as described in Methods. Briefly, reactivation of GdmCl denatured luciferase (2 μM) was induced by dilution 1:200 in 800 nM Hsc70 and 200 nM DnaJ or 1500 nM Hsp70 und 1000 nM DnaJ in renaturation buffer (10 mM MOPS, pH 7.8, 50 mM KCl, 5 mM ATP, 5 mM MgCl_2_, 5 μM BSA) in the presence of increasing concentrations of 9-aminoacridizinium bromide derivatives at 25 °C. After 2 h of renaturation the activity of luciferase was determined. IC_50_ value determinations were based on duplicate eight-point dose response curves. The mean IC_50_ and standard error of the measurements were calculated via nonlinear regression.

**Table 2:** Cytotoxicity of 1 and 3 against different human cell lines.

cell line	EC_50_value (μM)	
	1	3
HeLa	19.5 ± 2.5	35.8 ± 12.4
HEK-293	29.1 ± 1.1	22.6 ± 1.3
HT-29	71.6 ± 34.0	114.7 ± 52.4
SH-SY5Y	58.1 ± 4.6	70.0 ± 10.5

^a^EC_50_ values were determined as described in Methods.
