# Supplementary material for: A novel Hsp70 inhibitor prevents cell intoxication with the actin ADP-ribosylating Clostridium perfringens iota toxin
Source: Sci Rep. 2016 Feb 3;6:20301. doi: 10.1038/srep20301 (PMC4738285; doi:10.1038/srep20301)
Supplement: Supplementary Information [file srep20301-s1.pdf]

A novel Hsp70 inhibitor prevents cell intoxication with the actin ADP-ribosylating *Clostridium perfringens* iota toxin

Katharina Ernst[2*], Markus Liebscher[3*], Sebastian Mathea[3], Anton Granzhan[4], Johannes Schmid[2], Michel R. Popoff[5], Heiko Ihmels[4], Holger Barth[2**], Cordelia Schiene-Fischer[1,3**]

Supplementary information:

Supplementary figure S1.

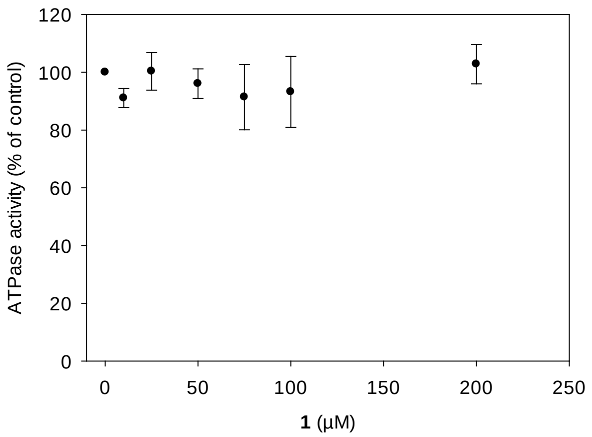

Figure S1. ATPase activity of Hsp70 in the presence of increasing concentrations of 1. Experiments were performed using 0.5 µM of Hsp70 in 40 mM HEPES buffer pH 7.6, 50 mM KCl, 11 mM $MgCl_2$, 1 mM ATP. Detection of the produced phosphate was detected according to Bartolommei *et al.*, 2013 (Bartolommei, G., Moncelli, M.R. & Tadini-Buoninsegni, F. A method to measure hydrolytic activity of adenosinetriphosphatases (ATPases). *PLOS ONE* **8**, e58615 (2013).).

Supplementary figure S2.

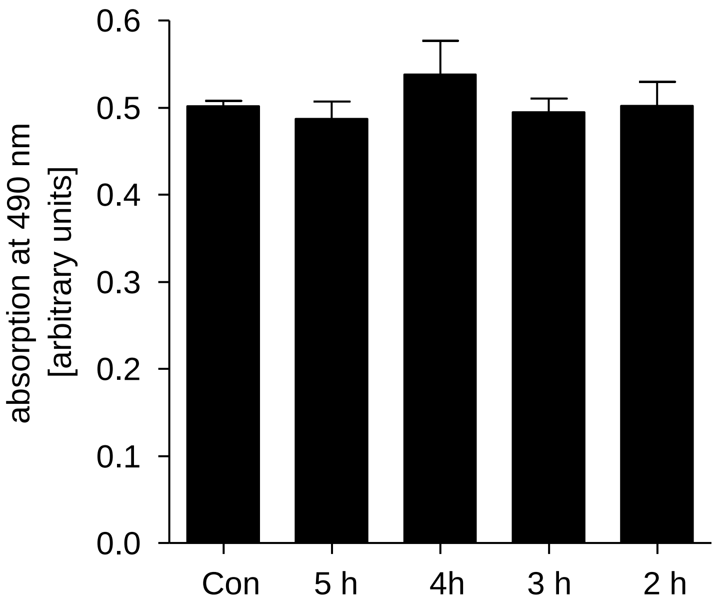

Figure S2. Compound 1 does not impair cell viability of Vero cells within 5 h of incubation. Vero cells were incubated at 37 °C with 100 µM of compound 1 for the indicated time periods or left untreated for control (con). Subsequently, the medium was removed and fresh medium containing MTS reagent (Promega) was added to the cells. After 1 h of incubation at 37 °C the absorption at 490 nm was measured. Values are given as mean as mean ± SD (n = 3).

Supplementary figure S3.

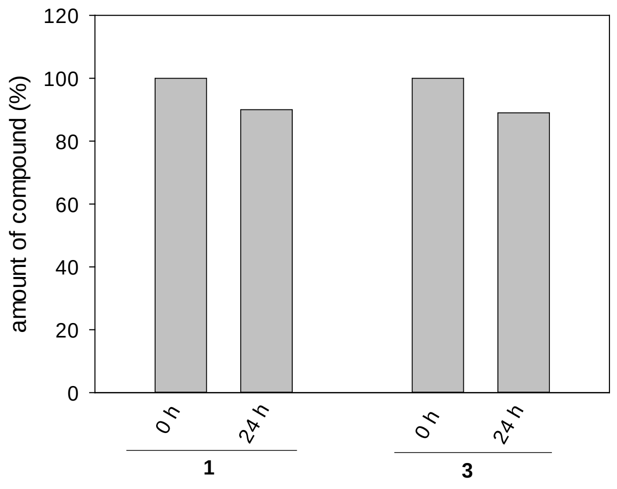

Figure S3. Metabolic stability of 1 and 3 in HeLa cell lysate. HeLa cells exposed to 100 µM of each acridizinium derivate **1** or **3** for 24 h were subsequently harvested and lysed. Cell lysates were analyzed by HPLC and mass spectrometry. Both compounds **1** and **3** were present at concentrations of about 5 µg/ml (18 µM) under identical conditions. Depicted is the percentage of the compounds remaining directly or 24 h after addition to the HeLa cells. After 24 h of incubation, 90% of the compounds **1** and **3** remained intact in HeLa cell lysate.

Supplementary figure S4.

a)

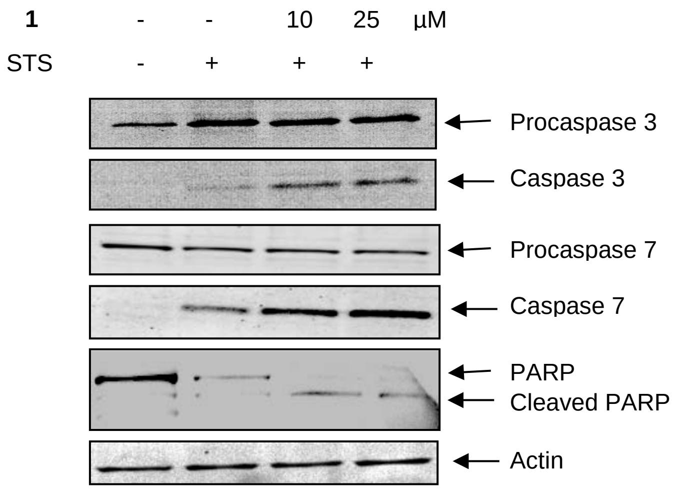

b)

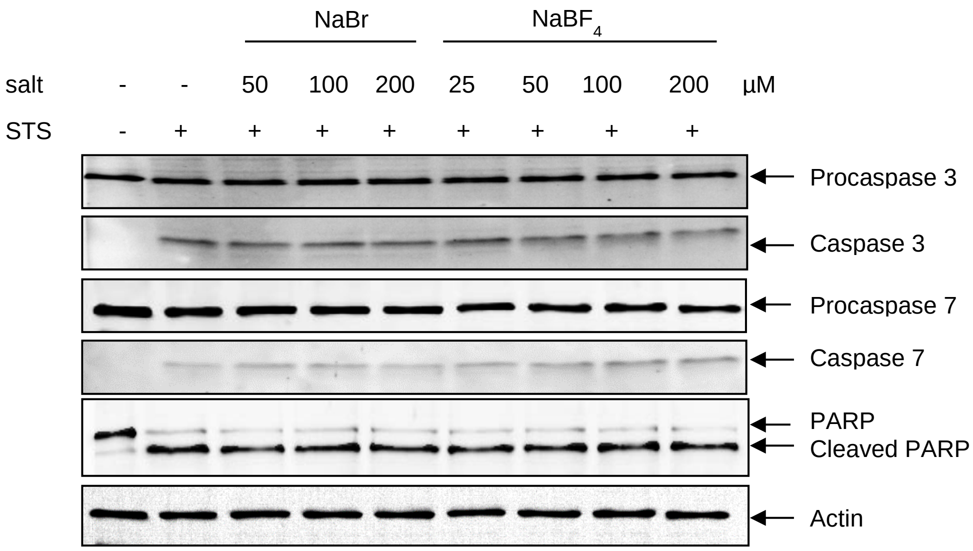

Figure S4. a) Synergistic activation of apoptosis by staurosporine and 1. Induction of apoptosis in HeLa cells by staurosporine was increased by low doses of **1**. Confluent HeLa cells were exposed to a combination of **1** and staurosporine. The combination of **1** and staurosporine increased the formation of caspase 3 and caspase 7 by proteolytic cleavage of caspase 3 and 7 proenzymes and the proteolytic cleavage of PARP indicative of apoptosis. Proteins of the cell lysates were resolved by SDS-PAGE and analyzed by immunoblotting Analysis of the proteolytic cleavage caspase-3 proenzyme, caspase-7 proenzyme, and of poly (ADP-ribose) polymerase (PARP). Staurosporine was applied at 0.5 µM, the concentration of **1** was 10 µM (+) or 25 µM (++). **b) NaBr and $NaBF_4$ do not affect apoptosis of HeLa cells.** Confluent HeLa cells were exposed to a combination of staurosporine and different concentrations of NaBr or $NaBF_4$. The addition of the salts in the presence of staurosporine had no effect on the formation of caspase 3 and caspase 7 by proteolytic cleavage of caspase 3 and 7 proenzymes and the proteolytic cleavage of PARP indicative of apoptosis. Proteins of the cell lysates were resolved by SDS-PAGE and analyzed by immunoblotting Analysis of the proteolytic cleavage caspase-3 proenzyme, caspase-7 proenzyme, and of poly (ADP-ribose) polymerase (PARP). Staurosporine was applied at 0.5 µM. Experiments were performed in triplicate.

Supplementary figure S5.

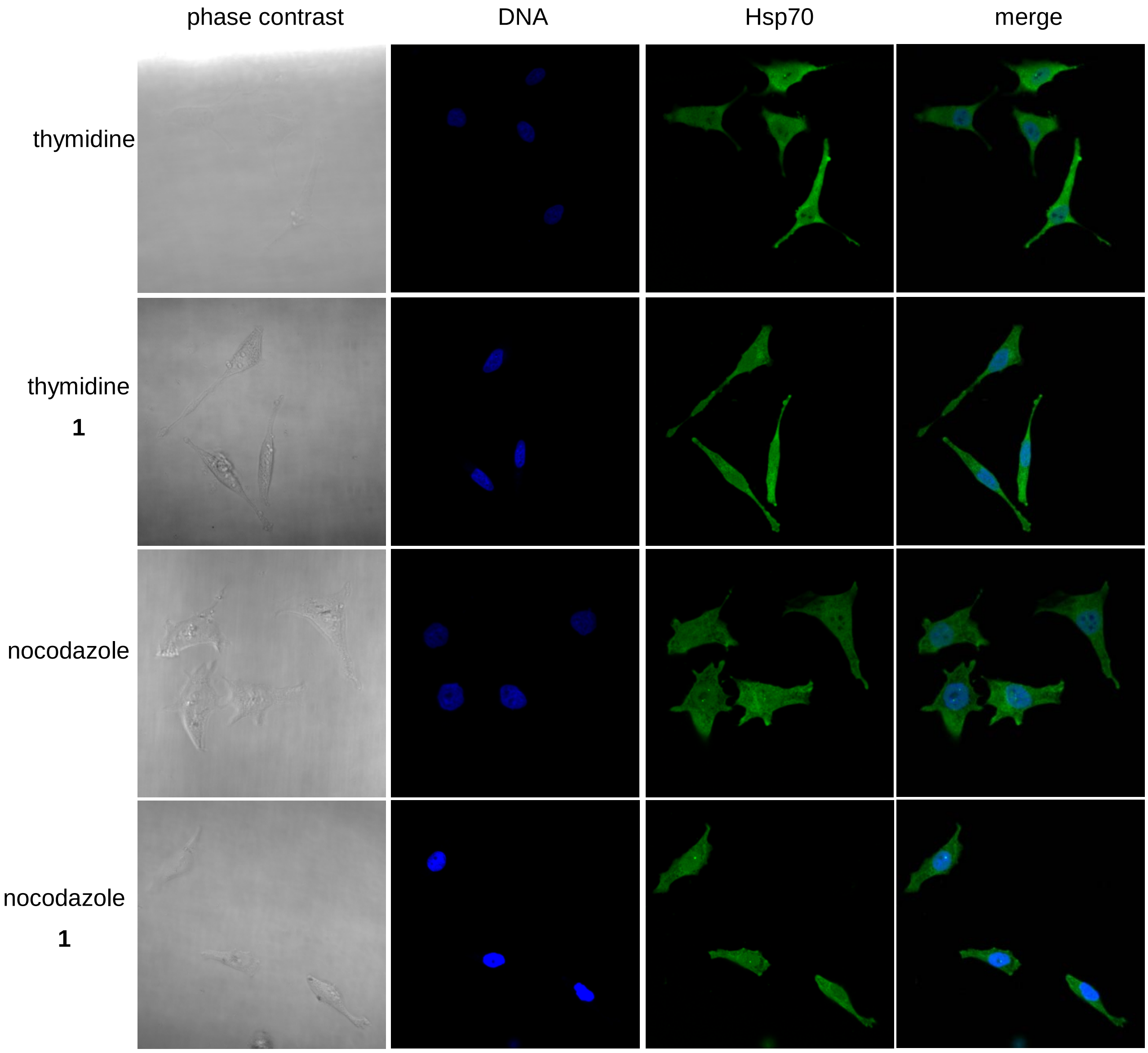

Figure S5. Localization of Hsp70 in HeLa cells in the G1/S or G2/M phase is not changed by 1. Cells were treated with thymidine or nocodazole for 18 h followed by additional application of 1 for 4 h. Cells were fixed and labeled with antibodies to Hsp70 (green). DNA was stained by DAPI (blue). Hsp70 localized diffusely distributed in the nucleus and cytoplasm both, in cells in the G1/S phase (thymidine) and in cells in the G2/M phase (nocodazole). Exposure of the cells to the acridizinium derivative **1** had no significant influence on Hsp70 distribution in the nucleus and cytoplasm.

Supplementary figure S6.

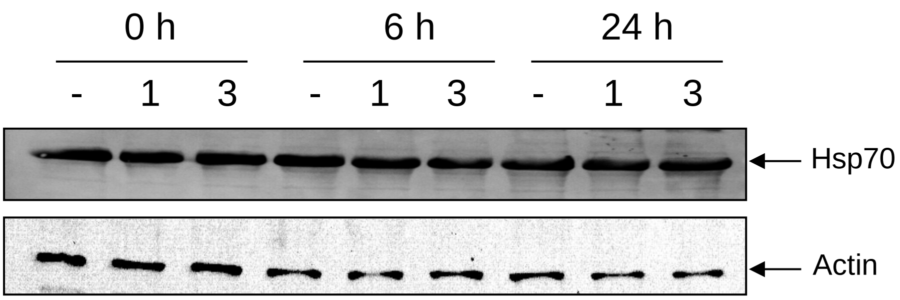

Figure S6. Hsp70 in HeLa cell lysate in the presence of 1 and 3 after 0, 6 or 24 h incubation. Approximately 60% confluent HeLa cells were grown in the presence of 25 µM of **1** or **3** or an equivalent volume of DMSO as a control. Cells were harvested after 0, 6 and 24 h. Cell lysates were analyzed for Hsp70 by Western blot analysis using anti-Hsp70 antibody and anti-Actin antibody as a loading control.
